# In Vitro Anticancer Properties of Table Grape Powder Extract (GPE) in Prostate Cancer

**DOI:** 10.3390/nu10111804

**Published:** 2018-11-20

**Authors:** Avinash Kumar, Melinee D’silva, Kshiti Dholakia, Anait S. Levenson

**Affiliations:** 1 Arnold & Marie Schwartz College of Pharmacy and Health Sciences, Long Island University, Brooklyn, NY 11201, USA; avinash.kumar@liu.edu (A.K.); melinee.dsilva@my.liu.edu (M.D.); kshiti.dholakia@my.liu.edu (K.D.); 2 College of Veterinary Medicine, Long Island University, Brookville, NY 11548, USA

**Keywords:** grape powder extract, prostate cancer, MTA1

## Abstract

Although the link between diet and cancer is complex, epidemiological data confirm that diet is a risk factor for prostate cancer and indicate a reduced prostate cancer incidence associated with a diet rich in vegetables and fruits. Because of the known protective effect of grape seed extract (GSE) against prostate cancer, we evaluated the effects of grape powder extract (GPE) on cell viability, proliferation, and metastatic capability. Importantly, we explored the possible novel mechanism of GPE through metastasis-associated protein 1 (MTA1) downregulation in prostate cancer, since our previous studies indicated resveratrol (Res)- and pterostilbene (Pter)-induced MTA1-mediated anticancer activities in prostate cancer. We found that GPE inhibited the cell viability and growth of prostate cancer cells only at high 100 μg/mL concentrations. However, at low 1.5–15 μg/mL concentrations, GPE significantly reduced the colony formation and wound healing capabilities of both DU145 and PC3M cells. Moreover, we found that GPE inhibited MTA1 in a dose-dependent manner in these cells, albeit with considerably less potency than Res and Pter. These results indicate that stilbenes such as Res and Pter specifically and potently inhibit MTA1 and MTA1-associated proteins compared to GPE, which contains low concentrations of Res and mainly consists of other flavonoids and anthocyanidins. Our findings support continued interest in GPE as a chemopreventive and anti-cancer agent against prostate cancer but also emphasize the unique and specific properties of stilbenes on MTA1-mediated anticancer effects on prostate cancer.

## 1. Introduction

Despite progresses in understanding the molecular mechanisms of prostate cancer (PCa), it is still the most frequently diagnosed cancer in men in the United States, specifically in recent years, in which life expectancy has increased. Most men acquire PCa during their lifetime because of risk factors such as age and diet. Dietary bioactive polyphenols with anti-inflammatory, antioxidant, and anticancer properties have been of intense interest for use as chemopreventive agents against PCa. Particularly, stilbenes such as resveratrol (*trans*-3,5,4’-trihydroxystilbene, Res) and its natural analogs including pterostilbene (*trans*-3,5-dimethoxystilbene, Pter), found in grapes and berries [1,2], have attracted attention as potential pharmacological approaches for primary and clinical chemoprevention of PCa [3,4,5,6,7,8,9,10,11,12,13,14,15,16].

However, only limited studies support separate polyphenol(s) use in human chemoprevention due to their low bioavailability and rapid metabolism [17,18,19]. Therefore, grape extract, which contains a mix of various polyphenols including stilbenes, might present improved pharmacokinetics and superior pharmacological potency to stilbenes alone and may hold greater potential as a natural product drug. Grape seed extract (GSE), which is actively available as a health food supplement, has been shown to have strong antioxidant capabilities [20], cardiac benefits [21,22], neurological effects [23], and cancer preventive and anticancer activities [24,25,26,27,28,29,30]. Particularly, it has been shown that GSE has anticancer effects against PCa in vitro and in vivo [24,28,29,31]. Importantly, specific signaling pathways were identified as GSE-regulated, including androgen receptor (AR)-mediated transcription of genes [24] and inhibition of the activation of extracellular signal-regulated kinase 1/2 (ERK 1/2) with associated apoptotic effects [28].

Our interest includes AR-independent pathways that play a role in the progression of PCa. One of these pathways is represented by an overexpression of metastasis-associated protein 1 (MTA1) and the subsequent activation of MTA1-mediated pro-oncogenic signaling associated with the progression of PCa to metastasis [3,11,12,13,16,32,33,34,35,36]. Clinical studies have demonstrated the correlation of high MTA1 expression in prostate tissues with aggressive clinicopathological characteristics of tumors, signifying MTA1 as a potential therapeutic target in PCa. Therefore, we have intensively investigated and reported on the MTA1-mediated anticancer properties of Res and Pter in PCa in vitro and in vivo [3,4,7,8,11,12,13,14,16].

The present study aimed to investigate the anticancer efficacy and MTA1 targeting ability of grape powder extract (GPE) in PCa cell lines. Grape powder extract consists of seven flavonoids, including resveratrol and three anthocyanidins (Table 1) [37]. We performed cell-based assays with GPE in two PCa cell lines using Res and Pter as reference compounds. Our results indicate that GPE has anticancer and antimetastatic effects in PCa, while stilbenes such as Res and Pter have the strongest MTA1 inhibitory action. Therefore, grape extract enriched for stilbenes through unique extraction procedures may represent an effective dietary agent for chemopreventive and therapeutic activity against PCa.

## 2. Materials and Methods

### 2.1. Compounds

Grape powder, which is a proportional representation of the different varieties of table grapes grown in California, was obtained from the California Table Grape Commission. The grape powder extract (GPE) was prepared and standardized as described previously [37] and was a generous gift from Dr. Richard van Breemen (Linus Pauling Institute, Oregon State University, Corvallis, OR, USA). Resveratrol and pterostilbene were purchased from Sigma-Aldrich (St. Louis, MO, USA) and were dissolved in dimethyl sulfoxide (DMSO) for the in vitro experiments.

### 2.2. Cell Culture 

Prostate cancer cells, DU145 and PC3M, were maintained in RPMI-1640 media containing 10% FBS in an incubator at 37 °C with 5% CO_2_ as described previously [3,6,8,9,10,11,12,13,14,15,16,33,34,36]. Cells were authenticated using short tandem repeat profiling at Research Technology Support Facility, Michigan State University.

### 2.3. MTT Assay

Cell viability of DU145 and PC3M cells was measured after treatment with GPE (2–200 µg/mL), Res (5–100 µM), and Pter (5–100 µM) using MTT assay (Sigma-Aldrich, St. Louis, MO, USA) as described previously [6,16]. Briefly, the cells were seeded in 96-well plates and treated with vehicle, GPE, Res, or Pter. Absorbance of the formazan was measured using BioTek Synergy-4 plate reader (BioTek, Winooski, VT, USA) after 72 h of treatment. The % cell viability was calculated assuming 100% viability in vehicle-treated (control) wells.

### 2.4. Proliferation Assay

DU145 and PC3M cells (2 × 10^3^) were seeded in a 35-mm cell culture dish. The media with appropriate compound (GPE, Res, or Pter) was changed every other day. The proliferation of the cells was determined by counting the cells every other day over a period of 10 days.

### 2.5. Colony Formation Assay

Colony formation assay was performed as described previously [33]. Briefly, cells (5 × 10^3^) were seeded in a 35 mm cell culture dish for a 21-day observation time. The media with appropriate compound was changed every other day. When colonies were freely visible (>50 cells/colony) in vehicle-treated dish, cells were fixed with formaldehyde and stained with 0.1% crystal violet solution. Colonies were visualized by imaging each dish using Amersham Imager 600 (GE Healthcare Bio-Sciences, Pittsburg, PA, USA). ImageQuant TL software (GE Healthcare Bio-Sciences, Pittsburg, PA, USA) was used for counting the number of colonies in each dish.

### 2.6. Wound-Healing Assay

Wound-healing assay was performed as described previously [33,36]. Briefly, 95% confluent cells seeded in 6-well plates were starved in low serum media (0.1% serum) overnight, after which three separate wounds were scratched across the well. The media with appropriate compound was changed every other day. The wound was imaged daily until the wounds of vehicle-treated (control) cells were completely closed using the EVOS XL Core microscope (ThermoFisher Scientific, Waltham, MA, USA). Wound area was calculated using the ImageJ software (NIH, Bethesda, MD, USA). % wound area was quantitated assuming 100% for vehicle-treated cells at 0 h.

### 2.7. Western Blot 

Western blot analysis was carried out as previously described [33,36]. Briefly, cells were treated with various concentrations of GPE (25–200 µg/mL), Res (50 µM), or Pter (50 µM) for 24 h, and total protein was extracted. Protein concentration was measured using Bio-Rad protein assay reagent (Bio-Rad Laboratories, Hercules, CA, USA). An equal amount of protein was resolved in 10–15% gels and transferred to a polyvinylidene difluoride (PVDF) membrane. After blocking the membranes for non-specificity, they were probed with MTA1 (1:2000), p21 (1:1000), cleaved caspase 3 (1:1000), PTEN (1:1000), Cyclin D1 (1:1000), and pAkt (1:1000) (Cell Signaling Technology, Danvers, MA, USA) primary antibodies. β-actin antibody (1:2500) (Santa Cruz, Dallas, TX, USA) was used as a loading control. Signals were visualized using enhanced chemoluminescence. Densitometry was performed using Image J software (NIH, Bethesda, MD, USA).

### 2.8. Statistical Analysis

The differences between the groups were analyzed by one-way analysis of variance (ANOVA). All statistics were performed using GraphPad Prism 7 software (GraphPad Software, La Jolla, CA, USA). The statistical significance was set as *p* < 0.05. All data are cumulative of at least three independent experiments.

## 3. Results

**Figure 1:**
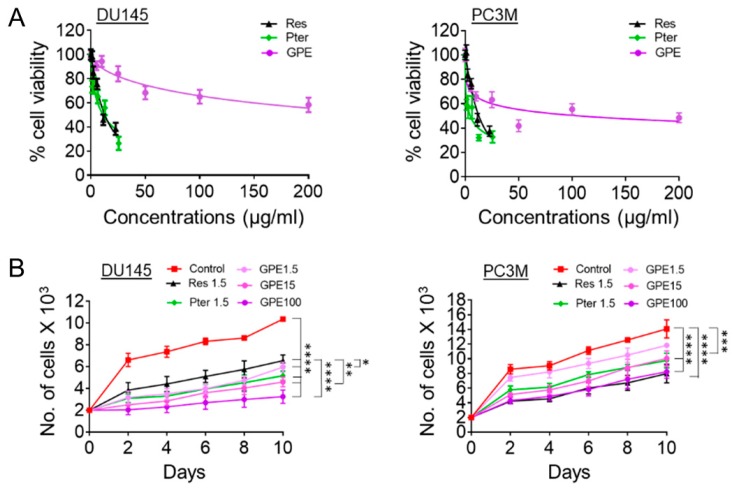
Effects of GPE on cell viability and cell proliferation. (**A**) Cell viability analysis of DU145 (left) and PC3M (right) prostate cancer cells treated with GPE (2–200 µg/mL), Res (1.14–22.8 µg/mL), and Pter (1.28–25.6 µg/mL). Data represent the mean ± scanning electron microscopy (SEM) of three independent sets of experiments. (**B**) Proliferation assay of DU145 (left) and PC3M (right) cells after treatment with GPE (1.5; 15; 100 µg/mL) and Res and Pter (1.5 µg/mL) for 10 days. Data represent the mean ± SEM of three independent sets of experiments. * *p* < 0.05, ** *p* < 0.01, *** *p* < 0.001, and **** *p* < 0.0001 (one-way analysis of variance (ANOVA)) were assessed as significant differences between treated and Ctrl vehicle cells.

The cytotoxic effects of GPE were evaluated and compared to those of Res and Pter by MTT cell viability assay in DU145 and PC3M aggressive prostate cancer cell lines. The cells were treated with various concentrations of GPE (5–200 μg/mL), Res (5–100 µM or 1.14–22.8 μg/mL), and Pter (5–100 µM or 1.28–25.6 μg/mL) for 72 h. As shown in Figure 1A, treatment with GPE had a modest cytotoxic effect even at high 200 µg/mL dose, whereas Res and Pter significantly inhibited the DU145 and PC3M cells’ viability in relatively low concentrations. The IC_50_ values of Res and Pter in DU145 and PC3M cells were in accordance with our previous reports [10], while GPE showed very low activity in both cell lines, with IC_50_ values of 107 μg/mL in DU145 cells. We were not able to determine IC_50_ values for GPE doses (5–200 μg/mL) used in PC3M cells. To further investigate the inhibition of cell proliferation by GPE, we performed cell-counting assay upon treatment with compounds. We counted cell numbers every other day for 10 days and found that GPE significantly inhibited cell proliferation compared to control untreated cells in all concentrations tested in both DU145 and PC3M cells (Figure 1B). In DU145 cells, higher concentrations of GPE (15 and 100 μg/mL) were significantly more potent at inhibiting cell growth (*p* < 0.01; *p* < 0.0001) than Res but not Pter. In PC3M cells, GPE at high doses (15 and 100 μg/mL) as well as Res and Pter showed significant differences compared to control vehicle-treated cells (*p* < 0.001; *p* < 0.0001).

**Figure 2:**
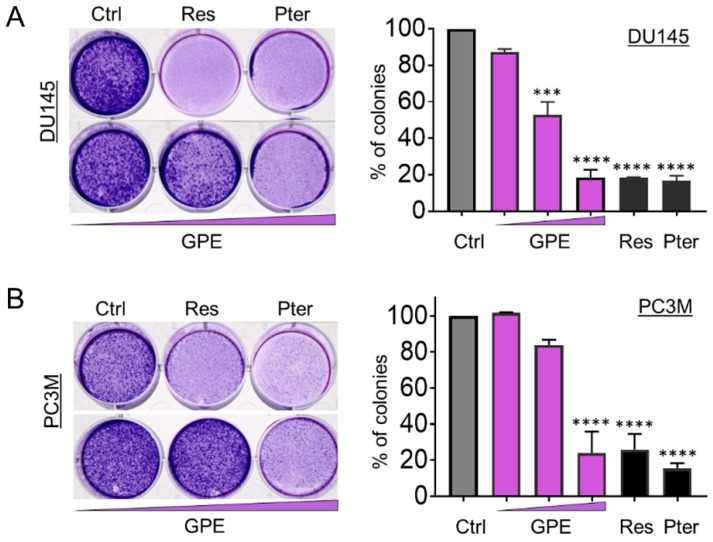
GPE reduces colony formation in DU145 and PC3M prostate cancer cells. Representative images of colony formation ability of (**A**) DU145 and (**B**) PC3M cells after treatment with GPE (1.5; 15; 100 µg/mL) and Res and Pter (1.5 µg/mL). Data represent the mean ± SEM of three independent experiments with duplicate wells. *** *p* < 0.001 and **** *p* < 0.0001 (one-way ANOVA) were assessed as significant differences between treated and Ctrl vehicle cells.

**Figure 3:**
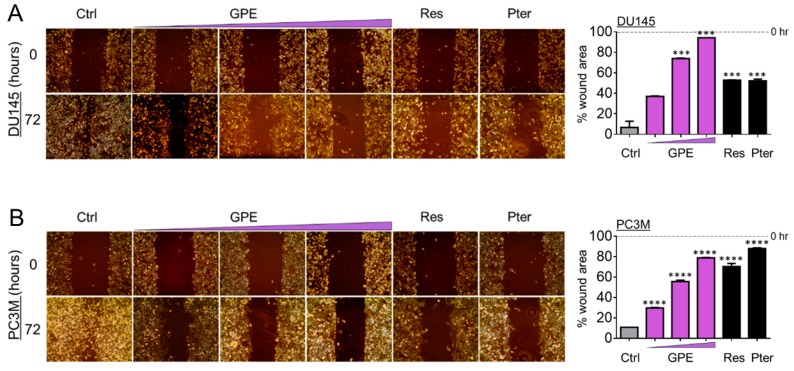
GPE reduces migration of DU145 and PC3M prostate cancer cells. Representative images of migration ability of (**A**) DU145 and (**B**) PC3M cells after treatment with GPE (1.5; 15; 100 µg/mL) and Res and Pter (1.5 µg/mL). Right: quantitation of wound widths, as % wound area is shown for each cell line. Data represent the mean ± SEM of six separate wounds and three independent experiments. *** *p* < 0.001 and **** *p* < 0.0001 (one-way ANOVA) were assessed as significant differences between treated and Ctrl vehicle cells.

To characterize the anti-metastatic capacity of GPE, we examined the effect of GPE on the colony formation and wound healing in DU145 and PC3M cells. As shown in Figure 2A, colony-forming ability was decreased in DU145 cells, with an increased GPE concentration gradient reaching significance at 15 μg/mL (*p* < 0.001) and 100 μg/mL (*p* < 0.0001). In more aggressive PC3M cells, GPE treatment at only a high (100 μg/mL) dose caused a significant reduction of colony-forming ability (*p* < 0.0001) (Figure 2B). However, these results indicated that only the effects of the highest concentration of GPE (100 μg/mL) were comparable with the effects of Res and Pter alone at a relatively low dose (1.5 μg/mL). After this, a wound-healing assay was performed in order to measure the effects of GPE treatment on tumor cell migration. The migration of DU145 and PC3M cells was decreased with an increased GPE concentration gradient (Figure 3A,B). As shown in Figure 3A, reduction in the cell migration of GPE-treated DU145 cells was highly significant (*p* < 0.001) in a dose-dependent manner, compared to control vehicle-treated cells. Interestingly, GPE-treated PC3M cells, which are characterized as more aggressive than DU145 cells, were affected in their migration capabilities by GPE treatment stronger than DU145 cells. These results indicated that GPE could reduce migration of aggressive prostate cancer cells in a dose-dependent manner starting at as low as 1.5 μg/mL dose. Once again, Res and Pter also showed strong ability to reduce migration of prostate cancer cells at 1.5 μg/mL dose.

**Figure 4:**
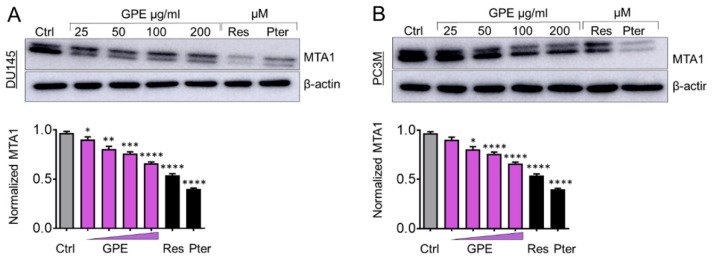
GPE inhibits MTA1 protein expression in a dose-dependent manner in DU145 and PC3M prostate cancer cells. Immunoblots of MTA1 expression in (**A**) DU145 and (**B**) PC3M cells (top panels). β-actin was used as a loading control. Western blots were repeated three times and representative blots are shown. Quantitation of immunoblot signals (lower panels). * *p* < 0.05, ** *p* < 0.01, *** *p* < 0.001, and **** *p* < 0.0001 (one-way ANOVA) were assessed as significant differences between treated and Ctrl vehicle cells.

**Figure 5:**
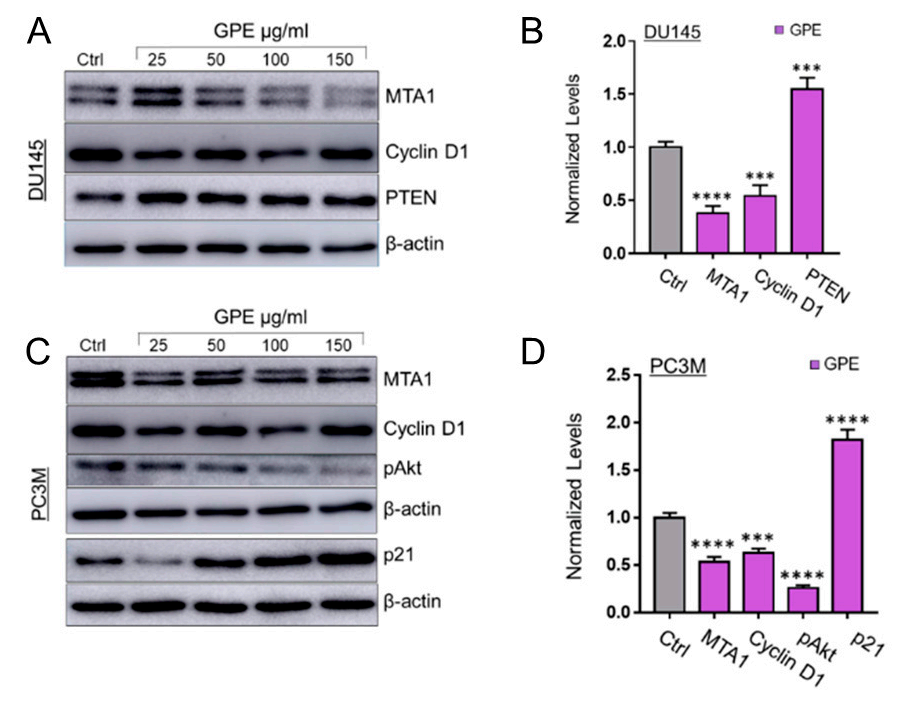
Effects of GPE treatment on MTA1-associated protein levels. (**A**) Immunoblots of MTA1, Cyclin D1, and PTEN expression, in DU145 cells treated with GPE. β-actin was used as a loading control. (**B**) Quantitation of immunoblot signals of MTA1-associated proteins, Cyclin D1, and PTEN, in cells treated with 150 μg/mL GPE. (**C**) Immunoblots of MTA1, Cyclin D1, pAkt, and p21 expression, in PC3M cells treated with GPE. β-actin was used as a loading control. (**D**) Quantitation of immunoblot signals of MTA1-associated proteins, Cyclin D1, pAkt, and p21, in cells treated with 150 μg/mL GPE. Western blots were repeated three times for each protein, and representative blots are shown. *** *p* < 0.001 and **** *p* < 0.0001 (one-way ANOVA) were assessed as significant differences between 150 μg/mL GPE-treated vs. Ctrl vehicle-treated cells.

We have shown previously that Res and Pter inhibit MTA1-mediated PCa progression in vitro and in vivo [3,7,8,11,13,16]. Therefore, we next measured the effect of GPE treatment on MTA1 protein expression in DU145 and PC3M cells. Cells were treated with GPE at various concentrations (25–200 μg/mL) and with Res and Pter (50 μM~12 μg/mL) for 24 h, after which the total protein was isolated and a western blot was performed. As indicated in Figure 4A,B, GPE downregulated MTA1 in a dose-dependent manner in DU145 cells and PC3M cells, respectively, but with considerably less potency than Res and Pter. We also sought to investigate the effect of GPE on certain MTA1-associated proteins in DU145 and PC3M cells, which we previously identified using ChIP-Seq [11]. We have shown that MTA1 directly regulates CyclinD1 and pAkt and negatively associates with PTEN [11,13]. As seen in Figure 5A,B, there was downregulation of Cyclin D1 and a slight upregulation of PTEN in DU145 cells treated with GPE. pAkt levels were affected by GPE treatment in PC3M cells (Figure 5C,D). Because p21 expression represents an important biomarker of apoptosis, we examined its expression after treatment with GPE. Results show that p21 was induced in a dose-dependent manner upon GPE treatment in PC3M cells (Figure 5C,D).

## 4. Discussion

Numerous studies have demonstrated that diet is a risk factor for PCa and consumption of plant foods may reduce the incidence of PCa [38,39,40]. Dietary phytochemicals such as quercetin, curcumin, genistein, selenium, resveratrol, and pterostilbene have been shown to possess anti-inflammatory, antioxidative, cardioprotective, and anticancer activities [4]. These polyphenols individually act through various genetic and epigenetic mechanisms to control numerous biochemical and molecular pathways, influencing cell growth, differentiation, cell cycle, senescence, apoptosis, epithelial-to-mesenchymal transition, and metastasis. While it is necessary to understand the mechanisms of action for individual bioactive molecules, the composition of dietary sources such as fruits or vegetables consists of various polyphenols, represented by different classes of phytochemicals that provide health benefits in unison. However, at the molecular level, the interaction of these molecules may cause the activation or inhibition of certain molecular pathways.

Published studies have demonstrated the prostate anticancer activity of GSE, which is comprised of high phenolic content. However, the total content might differ with possible active compounds depending on the method of extraction [28]. In the present study, we used grape powder extract (GPE), which is an organic solvent extract of freeze-dried whole table grapes. The varieties of grapes include a proportional representation of table grapes grown in California. Most table grapes grown in California are seedless, but some seeded grapes are produced and were included. After freeze drying, the grape skins and seeds remained and were ground to produce powder.

In this study, we demonstrated that GPE treatment caused growth inhibition and reduced the colony formation and migration ability of DU145 and PC3M prostate cancer cells. Less aggressive DU145 cells showed more sensitivity to the antiproliferative and anti-colony formation effects of GPE than to that of the PC3M cells. Because of our long-standing interest in targeting MTA1 signaling, we asked whether GPE acts through inhibition of MTA1 oncogenic protein in prostate cancer, as we have shown for Res and Pter, which has significant MTA1-mediated anticancer effects [3,7,8,11,13,16]. Our results suggest that although GPE downregulated MTA1 protein levels in a dose-dependent manner (p.6. 11–1128), the inhibition was not significant, possibly due to low nanomolar concentration of Res in GPE. As expected, Res and mainly Pter alone at 50 μM dose showed a marked inhibition of MTA1, suggesting a unique stilbenes–MTA1 relationship.

Taken together, GPE demonstrated anticancer effects in prostate cancer cell lines. Because of strong evidence for the involvement of MTA1 signaling in all stages of PCa progression, we explored a chance of MTA1 inhibition by GPE. Since we detected more potent MTA1 inhibition by stilbenes compared to mixture of polyphenols in GPE, there is a possibility that stilbenes and no other phytochemicals directly bind to MTA1 (unpublished data). Whether or not combination of various stilbenes will be most effective at inhibiting MTA1 and MTA1-guided signaling in PCa is unknown and remains to be elucidated.

In summary, our preclinical in vitro findings support continued interest in GPE as an anticancer agent against PCa. However, in vivo studies and especially clinical trials are needed to explore the chemopreventive and therapeutic effects of GPE. Two recent clinical trials of muscadine grape skin extract containing ellagic acid, quercetin, and resveratrol in men with biochemically recurrent PCa showed the safety of two different doses (500 mg and 4 g) but no significant differences in predictive biomarkers [41,42]. Nevertheless, recent advances in personalized medicine are very promising and may make the translational application of chemoprevention by natural products a distinct possibility in target-stratified patient populations.

## Figures and Tables

**Table 1:** Liquid Chromatography with tandem mass spectrometry (LC-MS/MS) analysis of the GPE (grape powder extract) and chemical structures of its compounds.

Class	Compound	Chemical Structure	Content (ppm)
**Phenols**	Catechin	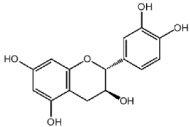	4014
	Epicatechin	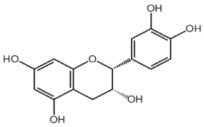	1268
**Flavonols**	Quercetin	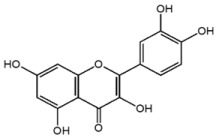	3429
	Kaempferol	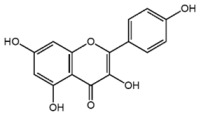	429
	Isorhamnetin	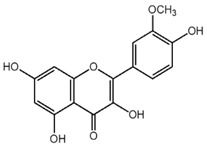	346
	Taxifolin	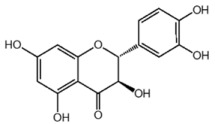	656
**Stilbenes**	Resveratrol	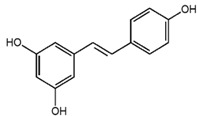	88.5
**Anthocyanins**	Cyanidin	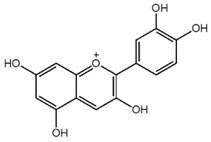	508
	Peonidin	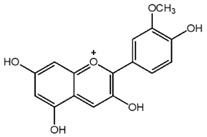	5034
	Malvidin	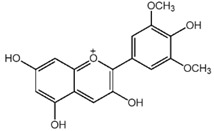	2811
